# Editorial: Recent advances in oral medicine in dogs and cats

**DOI:** 10.3389/fvets.2025.1753541

**Published:** 2026-01-13

**Authors:** Jamie Anderson, Maria Soltero-Rivera

**Affiliations:** 1 Sacramento Veterinary Dental Services, Rancho Cordova, CA, United States; 2 Department of Surgical and Radiological Sciences, University of California, Davis, Davis, CA, United States

**Keywords:** canine, feline, oral medicine, oral pathologies, veterinary

This series is the first to focus on oral and maxillofacial medicine challenges and advances in veterinary medicine practice. Discussion and inquiry into the discipline of veterinary oral and maxillofacial medicine is fundamental, timely, and necessary. Oral and maxillofacial medicine specializes in the diagnosis and management of diverse disorders of the oral maxillofacial region, including mucosal lesions, immunologic oral diseases, and insidious conditions such as cancers. This field also addresses microbiome-related disease such as periodontitis, oral-facial pain, non-surgical temporomandibular joint conditions, and salivary gland disorders. Moreover, it manages medically complex veterinary patients, with at times multiple co-morbidities, requiring oral and maxillofacial care. The growing field of veterinary oral and maxillofacial medicine, enhanced by recent breakthroughs in transcriptomic and single-cell resolution analysis, as well as targeted therapies, is set to transform the understanding and treatments of oral and maxillofacial disorders in animals. These cases pose challenges for veterinarians, requiring greater exposure to ensure precise diagnosis and effective management.

Recognizing the critical role of oral and maxillofacial medicine within the American Veterinary Dental College, and informed by recent survey results, we aim to address a notable gap: 71% of dental specialists reported significant frustration with the current lack of knowledge in this essential discipline. If such a deficit exists among specialists, the primary-care veterinary community likely faces an even greater need for accessible education and training in oral and maxillofacial medicine. Many of these disorders are characterized as idiopathic, highlighting the need for detailed description and understanding of their pathogenesis.

*Recent Advances in Oral Medicine in Dogs and Cats*, with 34K views, has successfully exposed primary care practitioners and specialists to the evolving landscape of oral and maxillofacial medicine. Through a focus on case-based clinical insights, review articles, and advanced clinical research, this Research Topic illuminated the field's intricacies, emphasizing cutting-edge diagnostic techniques and targeted therapeutic approaches. As a result, authors have not only helped close knowledge gaps but also raised awareness of oral and maxillofacial medicine as a distinctive subspecialty within Veterinary Dentistry. Collaborations with the American Academy of Oral Medicine have fostered the success of this special series and shared One Health objectives. Finally, leveraging Frontiers in Veterinary Science's renowned reputation, especially within the Veterinary Dentistry and Oromaxillofacial Surgery section, this series has proven to be a pioneering initiative, addressing a critical need in the field and establishing itself as the first of its kind.

To begin, an excellent review contribution by Dosenberry et al., in “*An update on oral manifestations of systemic disorders in dogs and cats*,” set the stage for discussion of veterinary oral medicine in the context of systemic health.

Focusing on immunoinflammatory disorders such as canine periodontitis and feline chronic gingivostomatitis yielded exceptional contributions to this edition. Gawor et al. conducted an open-label study titled “*Cathepsin K Inhibition by VBX1000 Alleviates Canine Periodontitis*.” The trial evaluated the safety and efficacy of the novel Cathepsin K inhibitor VBX1000 in 20 client-owned dogs with periodontal disease. Results indicate that VBX1000 is well tolerated and represents a promising therapeutic option for mild-to-moderate canine periodontitis. Feline chronic gingivostomatitis (FCGS) is a complex inflammatory and immune-mediated oral disease with elusive etiology and challenging treatment outcomes. Because FCGS had been anecdotally proposed as a pre-malignant oral disorder, Tsugawa et al. conducted a retrospective study titled “*Co-occurrence of Feline Chronic Gingivostomatitis and Oral Squamous Cell Carcinoma in 4 Cats (2014–2024)*.” Among 221 cases from two veterinary teaching hospitals, only four showed concurrent FCGS and oral squamous cell carcinoma (SCC), suggesting that FCGS is not a predisposing factor for SCC.

Recent advances in regenerative medicine have expanded the application of mesenchymal stromal cell (MSC) therapy beyond inflammatory conditions such as FCGS into oncology. The successful use of MSCs in FCGS provided a rationale to explore allogeneic feline umbilical cord-derived mesenchymal stromal cells (fUC-MSCs) in an adult cat with oral SCC. Park and Song reported this case in their article, “*Case Report: Allogeneic Feline Umbilical Cord-Derived Mesenchymal Stem Cell Transplantation for Feline Oral Squamous Cell Carcinoma*.” Although the clinical outcome was poor, the study suggests that fUC-MSCs may offer short-term benefits, including pain relief and transient tumor control, in feline oral SCC. These findings highlight the emerging potential of regenerative medicine as a complementary strategy in veterinary oncology.

Two recent case series examined osteonecrosis of the jaw, including a canine-focused study titled “*A Case Series and Review of Canine Idiopathic Osteonecrosis of the Jaw*” by Rossi and Anderson. Expanding on previous knowledge, this series of 10 dogs showed that lesions most often involved the caudal maxilla and the ipsilateral zygomatic arch, and that dental surgery was not always a preceding factor in lesion development. A feline-focused study titled “*A Retrospective Case Series on Bisphosphonate-Related Osteonecrosis of the Jaw in 20 Cats*” by Hatunen et al. highlight an emerging concern in veterinary oral medicine. As monoclonal antibodies and antiresorptive agents such as bisphosphonates gain wider use in cats—for conditions ranging from idiopathic hypercalcemia to tumor-associated bone resorption—clinicians must remain vigilant for medication-related osteonecrosis of the jaw (MRONJ), a well-recognized complication in humans. This sentinel report serves as an essential reminder of the need for cautious use and careful dental management in feline patients receiving these therapies.

Two articles in this Research Topic highlight advances in salivary gland research and diagnostics that expand the reach of veterinary oral and maxillofacial medicine. Story et al. describe the first well-characterized case of suspected Sjögren's-like disease in a dog, integrating imaging, histopathology, and immunohistochemistry to establish a diagnostic framework for autoimmune sialadenitis. Additionally, a timely mini review by Schroers and Meyer-Lindenberg provides a comprehensive overview of the diagnostic and translational potential of saliva testing in cats, emphasizing its utility beyond infectious disease detection to encompass dental, allergic, and metabolic disorders. The authors highlight saliva as a minimally invasive, information-rich biofluid with applications in both feline and comparative oral medicine. By consolidating evidence from 225 studies, this work defines saliva as an emerging diagnostic frontier, offering a foundation for cross-species approaches that link oral, systemic, and zoonotic health in veterinary patients.

Through its collection of case-based clinical insights, review articles, and advanced clinical research, this Research Topic illuminates the depth and breadth of veterinary oral and maxillofacial medicine, emphasizing innovations in diagnosis, therapeutics, and translational relevance. Collectively, these contributions not only help close critical knowledge gaps but also elevate oral and maxillofacial medicine as a distinctive and evolving subspecialty within veterinary dentistry. Because opportunities for training in Veterinary Oral and Maxillofacial Medicine are minimal, we are committed to promoting and fostering continued advancement in the field, recognizing that strong mentorship and academic collegiality are essential to success.

